# Acute and Repeated Dose 28-Day Oral Toxicity Study of the Aqueous Extracts from the Leafy Stem and Fruit of *Pedalium murex* D.Royen EX.L in Wistar Rats

**DOI:** 10.1155/2023/2962905

**Published:** 2023-07-21

**Authors:** Gérard Bessan Dossou-Agoin, Maxime Machioud Sangaré-Oumar, Téniola Isabelle Sacramento, Mariette Sindété, Egnon Jacques Hougbénou-Houngla, Nounagnon Darius Tossavi, Simon Azonbakin, Adam Gbankoto

**Affiliations:** ^1^Experimental Physiology and Pharmacology Laboratory, Faculty of Sciences and Technology, University of Abomey-Calavi, Abomey-Calavi, Benin; ^2^School of Management and Operation of Livestock Systems, National University of Agriculture, Ketou, Benin; ^3^Histology, Biology of Reproduction, Cytogenetics and Medical Genetics Laboratory, Faculty of Health Sciences, University of Abomey-Calavi, Abomey-Calavi, Benin

## Abstract

**Background:**

*Pedalium murex* (*P. murex*) is used in folk medicine for treatment of male infertility. However, scientific data on its safety are limited.

**Objective:**

This study was carried out to assess the acute and repeated dose 28-day oral toxicity of the aqueous extracts from *P. murex* leafy stem and fruit in Wistar rats.

**Methods:**

The acute toxicity test was performed according to the line 423 of the Organization for Economic Cooperation and Development (OECD) guidelines. The rats were randomly divided into three groups (*n* = 3). The control group received distilled water, while the experimental groups were given at a single dose, 5000 mg/kg of each extract. The repeated dose 28-day oral toxicity was performed according to the line 407 of the OECD guidelines. 35 rats divided into 7 groups of 5 male rats each were daily treated for 28 days with each extract at 200 mg/kg, 400 mg/kg, and 800 mg/kg, respectively. The in-life parameters were recorded during the follow-up. At the end of this study, organ weights, hematology, biochemistry, and histology parameters were analyzed.

**Results:**

In the acute oral toxicity test, there was no morbidity or mortality related to the treatments. Both extracts belong therefore to category 5 of the globally harmonized system (GHS) of classification. In the repeated dose 28-day oral toxicity test, both extracts did not alter animal's behavior. However, both extract administration led to proteinuria and renal damages.

**Conclusion:**

*P. murex* leafy stem and fruit aqueous extracts exhibited potential nephrotoxicity. Therefore, care should be taken when they are used over an extended period.

## 1. Introduction

Infertility is defined by the World Health Organization (WHO) as the inability to conceive after at least 12 months of regular unprotected sexual intercourse [1]. Male factor is responsible for about 50% of infertility cases [2]. In 30% to 40% of cases known as idiopathic, the etiology of male infertility is not known [3].

Idiopathic male infertility treatment relies on assisted reproductive techniques and empirical medical therapy [4]. The assisted reproductive technique cost makes it unaffordable to most of the couples [5]. The empirical medical therapy is based on low quality evidence and contradictory findings [6, 7]. This situation led infertile men to search for affordable, effective, and safe alternatives like phytotherapy [8].

Phytotherapy is the primary source of healthcare for about 80% of people in less developed countries [9]. Several medicinal plants are used to treat male infertility, including *Pedalium murex* (*P. murex*) [10]. The common misconception on medicinal plant safeness favors their consumption despite the risks of toxicity inherent in their overdose or long-term use [11].

*P. murex* belongs to the Pedaliaceae family and is commonly called “azizafen” in Benin. *P. murex* is used as aphrodisiac, to improve appetite and useful to treat asthma, gonorrhea, heart troubles, urinary discharges, skin diseases, and impotency [12]. The mucilaginous liquid resulting from shaking the leafy stem in water is used to treat seminal abnormalities and other male reproductive tract disorders. The dried powder of the fruit mixed with porridge is also used to alleviate male reproductive diseases [13]. In our previous study, preliminary phytochemical analysis of the aqueous extracts of *P. murex* leafy stem and fruit has revealed presence of flavonoids, tannins, alkaloids, coumarins, saponins, steroids, and lignans [14].

Different experimental models have confirmed the effectiveness of *P. murex* leafy stem or fruit folkloric use as aphrodisiac or male fertility booster [14–16]. Our prior study has also revealed the protective activity of *P. murex* leafy stem aqueous extract at 400 mg/kg against lead-induced testicular toxicity in Wistar rats [14]. Although phytochemicals could exhibit pharmacological effects, they could however be toxic [17].

A lot of data related to *P. murex* ethnomedicinal values and pharmacological activities are available [10]. However, there is a paucity of data on its safety and toxicity. It is the need to fill the gap on the potential side effects associated with the repeated oral treatment with the leafy stem and fruit of *P. murex* that prompted this work. Its aim was therefore to study the acute and repeated dose 28-day oral toxicity of the aqueous extracts of the leafy stem and fruit of *P. murex* in Wistar rats.

## 2. Materials and Methods

### 2.1. Plant Material

*P. murex* were harvested in June 2019 from Ahozon (6°37′65″N, 2°15′26″E, Benin Republic) and Ouidah (6°36′17″N, 2°05′58″E, Benin Republic). It has been identified at the Benin National Herbarium where a voucher specimen has been registered under the number YH 240/HNB. The leafy stem and fruit were washed thrice with tap water and dried under shade. The samples were powdered using a sample mill (Restch SM 2000).

### 2.2. Preparation of Extracts

50 g of the leafy stem and fruit powder was subjected to maceration in 500 ml of distilled water for 24 hours. The mixture was stirred continuously using an orbital magnetic stirrer. The filtrate was concentrated in the rotavapor (IKA-RV8) and then dried in an oven at 40°C for 2 hours. The extraction yields were equal to 15.56% ± 1.56 and 08.44% ± 0.92, respectively, for the leafy stem and fruit extract. The dried extracts were stored at 4°C for further use.

### 2.3. Animals

Nine-week-old male rats (168 ± 1.26 g) were purchased from the Husbandry of Histology, Biology of Reproduction, Cytogenetics, and Medical Genetics. The rats were housed in plastic cages and maintained at a room temperature of 22°–24°C with a 12 h dark/light cycle. The rats were fed with rodent chow (proteins: 18%, fat: 7.12%, carbohydrate: 14.14%, calcium: 1.16%, phosphorus: 0.80, and lysine: 0.82) and had *ad libitum* access to water.

### 2.4. Experimental Design

Male rats were used due to the fact that this study aims to evaluate the toxicity associated with treatment of male infertility with *P. murex*. This study follows a first one that has assessed the potential of the aqueous extracts from *P. murex* leafy stem and fruit in male infertility treatment [14].

#### 2.4.1. Acute Oral Toxicity

The acute oral toxicity of the extracts was assessed according to the OECD (Organization for Economic Cooperation and Development) line 423 guidelines [18]. Nine male rats were randomly divided into 3 groups of 3 rats each. The control group (Ctrl) received distilled water; groups LS5000 and FR5000 received, respectively, the leafy stem and fruit extracts at 5000 mg/kg. The test was performed at 5000 mg/kg to increase knowledge on toxicity related to *Pedalium murex* overdosing in human. Treatments were given via gavage. The rats were observed each day during 14 days for mortality, signs of acute toxicity, and behavioral changes. At the end of the experimental period, all animals were fasted overnight, weighed, and sacrificed under sodium thiopental anesthesia (50 mg/kg, IP). The liver, kidneys, stomach, spleen, heart, and lungs of the animals were removed and weighed. The liver and kidneys were fixed in 10% (v/v) formalin saline for histopathological analysis.

### 2.5. Repeated Dose 28-Day Oral Toxicity

It was performed according to the 407 guidelines of OECD [19]. 35 male Wistar rats were randomly divided into 7 groups of 5 rats each. The rats were daily treated via oral gavage with an intragastric canula. The control group received distilled water; LS200, LS400, and LS800 groups were treated with *P. murex* leafy stem at 200 mg/kg, 400 mg/kg, and 800 mg/kg, respectively; FR200, FR400, and FR800 groups were treated with the fruit of *P. murex* at 200 mg/kg, 400 mg/kg, and 800 mg/kg, respectively. The highest dose (800 mg/kg) was determined by multiplying the pharmacologically active dose by 2-fold intervals [14]. The rat behavior was observed during the follow-up. The observations included clinical signs such as tremors, convulsion, salivation, diarrhea, lethargy, and coma. In the last week of the study, animals were housed in metabolic cages and their urine was collected for 24 hours (12 hours in light cycle/12 hours in the dark cycle). Urinalysis was performed using test strips [20].

At the 28^th^ day, all animals were fasted overnight prior to necropsy. Rats were anesthetized with 50 mg/kg of sodium thiopental. Blood was collected by carotid exsanguination in EDTA and plain tubes for hematological and biochemical analysis. The liver, stomach, spleen, kidneys, lungs, heart, testes, and epididymis of the animals were weighed using an analytical scale (ESSE3). The liver, kidneys, and testes were fixed in 10% (v/v) formal saline for histopathological analysis.

### 2.6. Statistical Analyses

Data were expressed as the mean ± Standard Error on the mean (SEM). Data of acute toxicity were analyzed with the nonparametric Kruskal–Wallis and Dunn's multiple comparison tests. The data of the repeated dose 28-day oral toxicity test were analyzed through ANOVA followed by Dunnett's multiple comparison test. Values were considered statistically significant at *p* ≤ 0.05.

## 3. Results

### 3.1. Acute Oral Toxicity

#### 3.1.1. In-Life Observation

Both extracts did not cause morbidity or mortality. The treatment with fruit extract increased rat body weight at the end of the study when compared to the control group (Table 1). Both extracts increased the food intake when compared to the control group (Table 1). However, the body weight gain of both treated groups showed no change when compared to the control group (Table 1).

#### 3.1.2. Relative Organ Weight

The relative organ weight showed no change between the treated and control groups (Table 2).

#### 3.1.3. Histopathology

**Figure 1:**
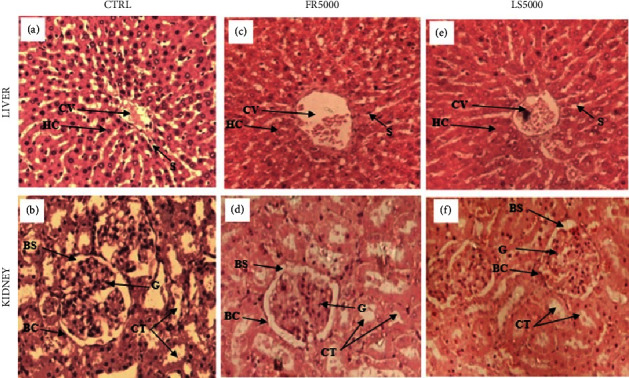
Liver and kidney-stained sections with hematoxylin and eosin (H&E, under ×20 magnification power). Representative sections of the liver and kidneys of control rats (a, b) and rats treated, respectively with 5000 mg/kg of fruit (c, d) and leafy stem extracts (e, f). Indicators: centrilobular vein (V), hepatic cord (HC), sinusoids (S), glomerular (G), Bowman's capsule (BC), Bowman's space (BS), and convoluted tubules (CT).

The gross examination of the liver and kidneys of the treated groups revealed no difference when compared to the control group. The treated groups showed normal liver and kidney histology when compared to the control group (Figure 1).

### 3.2. Repeated Dose 28-Day Oral Toxicity

#### 3.2.1. In-Life Observation

The treatment with both extracts did not lead to any toxic symptoms in rats. There were no behavioral differences between the treated and control groups. On the 3^rd^ week of the study, a higher mean body weight was noticed at 800 mg/kg in the leafy stem-treated group (Table 3). The group treated with the fruit extract depicted a lower mean body weight than the control group. This difference reached statistical significance at 400 mg/kg during the last week (Table 3). A significant decrease in the body weight gain was noticed after treatment with the fruit extract at 400 mg/kg (Table 3). The average food intake of the rats treated with the fruit extract at 400 mg/kg decreased significantly when compared to the control group during the 1^st^, 2^nd^, and 4^th^ weeks of the study (Table 4).

#### 3.2.2. Relative Organ Weights

At 400 mg/kg, the relative kidney weight increased significantly after treatment with both extracts. The relative weight of the heart and testes showed a significant increase at 400 mg/kg after treatment with the fruit extract (Table 5).

#### 3.2.3. Urinalysis

The urine from rats treated with the leafy stem showed proteinuria and leukocyturia at 400 mg/kg and 800 mg/kg (Table 6). The rats treated with the fruit extract exhibited proteinuria and hematuria at 400 mg/kg and 800 mg/kg (Table 6).

#### 3.2.4. Biochemistry

AST activity decreased significantly at 200 mg/kg, while it increased at 400 mg/kg in the leafy stem treated group. ALT activity increased at 400 mg/kg and 800 mg/kg in the leafy stem-treated group (Table 7). The fruit extract-treated rats showed a decrease in AST activity and an increase in ALT activity at 200 mg/kg. The leafy stem decreased glucose at 200 mg/kg, 400 mg/kg, and 800 mg/kg (Table 7). The leafy stem and fruit extracts increased the triglyceride amount at 400 mg/kg and at 800 mg/kg, respectively (Table 7).

#### 3.2.5. Hematology

Both extracts decreased the leukocyte count when compared to control, but statistical significance was reached only at 800 mg/kg. The leafy stem extract increased the platelet count at 200 mg/kg while decreasing it at 400 mg/kg and 800 mg/kg. The fruit extract led to a marked increase in the platelet count at 400 mg/kg and 800 mg/kg (Table 8).

#### 3.2.6. Histology

**Figure 2:**
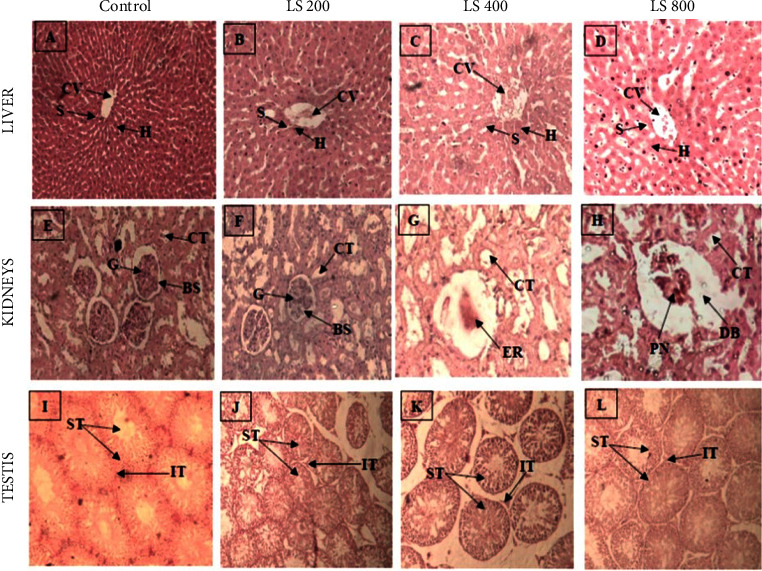
Photomicrographs of hematoxylin and eosin (H & E, under ×20 magnification power) stained sections of the liver (A–D), kidneys (E–H), and testis (I–L) from rats treated with distilled water and 200 mg/kg, 400 mg/kg, and 800 mg/kg of *P. murex* leafy stem aqueous extract for 28 days consecutively. Indicators: centrilobular vein (V), hepatic cord (H), sinusoids (S), glomerular (G), Bowman's space (BS), convoluted tubules (CT), seminiferous tubules (ST), interstitial tissue (IT), dilated Bowman's space (DB), pyknotic nucleus cells (PN), and empty renal corpuscle (ER).

**Figure 3:**
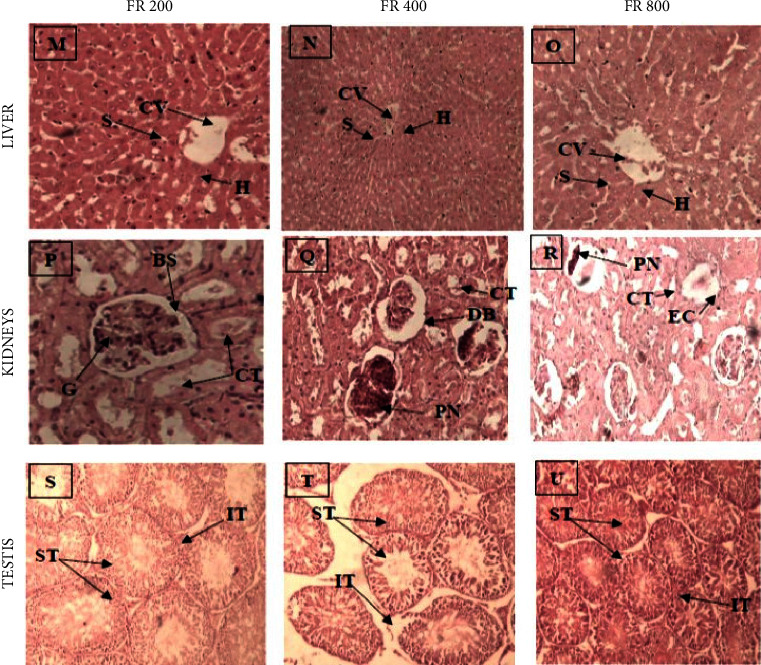
Photomicrographs of hematoxylin and eosin (H & E, under ×20 magnification power) stained sections of the liver (M–O), kidney (P–R), and testis (S–U) from rats treated with 200 mg/kg, 400 mg/kg, and 800 mg/kg of *P. murex* fruit aqueous extract for 28 days consecutively. Indicators: centrilobular vein (V), hepatic cord (H), sinusoids (S), glomerular (G), Bowman's space (BS), convoluted tubules (CT), seminiferous tubules (ST), interstitial tissue (IT), dilated Bowman's space (DB), pyknotic nucleus cells (PN), and empty renal corpuscle (ER).

Both extracts led to glomeruli shrinkage, karyopyknosis, and dilatation of Bowman's space at 400 mg/kg and 800 mg/kg (Figures 2 and 3). Moreover, several renal corpuscles depicted no glomerulus (Figure 3).

## 4. Discussion

The current treatment of idiopathic male infertility in orthodox medicine is unaffordable and ineffective [21, 22]. Therefore, many couples do not hesitate to use medicinal plants including *P. murex* despite their potential toxicity. This study aimed to investigate the acute and repeated dose 28-day oral toxicity of *P. murex* leafy stem and fruit aqueous extracts. The repeated treatment with both extracts revealed their potential nephrotoxicity.

Ideally, the toxicity of the xenobiotics to which people are exposed should be performed on humans. However, this experimental approach is unethical. Therefore, toxicity studies are preferentially performed on animals' models including the rat. The rat is often used due to its metabolic rate similarity to humans, its small size, short lifespan, and gestation time [23].

The acute toxicity of a chemical is estimated through its median lethal dose (LD_50_). In this study, the single administration of the aqueous extracts from the leafy stem and fruit of *P. murex* at 5000 mg/kg did not induce morbidity and mortality. Hence, both extracts belong to category 5 of the globally harmonized system (GHS) of classification. This finding agrees with prior data of Sharma et al. [15] and Gomathi et al. [24]. Our data revealed that the treatment with both extracts increased the food intake but did not modify the body weight gain. This finding could be linked to the type of food predominant nutrient. The chow given to the rat in this study was rich in dietary protein. But, according to Wu et al. [25], only the increment in food dietary fat was associated with the body weight gain in mice.

However, factors such as poor absorption and the first-pass effect may decrease extract bioavailability and mislead the appreciation of their toxicity [26]. Moreover, medicinal plants are often used for prolonged periods. Hence, it is important to assess their toxicity over long-term treatment.

The repeated dose 28-day oral toxicity study provides data on the health risks associated with repeated exposure to a test substance over a limited period of time [19]. To our knowledge, this study is the first that has evaluated the repeated dose 28-day oral toxicity of *P*. *murex* in rodents.

No behavioral changes, toxic signs, and mortality resulted from the treatment with the aqueous extracts from *P. murex* leafy stem and fruit. Body weight is used to monitor general health status [27]. The fruit extract induced a loss of body weight and decreased the body weight gain at 400 mg/kg (bw). Although the body weight loss has not reached statistical significance for other doses (200 mg/kg and 800 mg/kg), this decrease was higher than 5% when compared to the control group. According to Silva et al. [27], a body weight loss of 5% is predictive of organ injury. These data suggest that repeated treatment with the fruit of *P. murex* could be toxic.

Hematological parameters are the most sensitive to toxicants and have a high predictive value when data are extrapolated from animals to humans [28]. In this study, repeated treatment with both extracts showed a dose-dependent decrease in the leukocyte count. This finding could indicate that animals are exposed to stress factors or are highly vulnerable to a toxicant [29]. The leafy stem aqueous extract has led to a nondose-dependent variation in the platelet count. However, the fruit extract significantly increased the platelet count at 400 mg/kg and 800 mg/kg. Since a high platelet count increases the risk of thrombosis in humans, a more in-depth assessment through further studies of the effects of *P. murex* on coagulation is required to elucidate these findings.

Organ weight changes are often correlated with treatment effects [30]. The absolute organ weight changes with the animal body weight. Therefore, the relative organ weight is used to eliminate the bias associated with normal variation in animal growth [31]. In the present study, treatment with both extracts led to a nondose-dependent increase in the relative organ weight of the kidneys. This finding could therefore be incidental. However, the organ weight data should be always analyzed in an integrated way with data of biochemistry, hematology, and histology [29].

Nephrotoxicity is usually evaluated through urinalysis, serum biochemistry, and kidney histopathology. Blood urea and creatinine are the classic indices of the glomerular filtration rate (GFR). The increase in blood urea and creatinine above the reference range are inversely correlated to the GFR [32]. Both extract administration has not modified urea and creatinine levels. However, urinalysis has revealed proteinuria after treatment with both extracts. Furthermore, several glomerular damages in the kidneys were noticed after treatment with both extracts. Therefore, both extracts depicted potential nephrotoxicity. *Pedalium murex* nephrotoxicity could be due to its steroidal saponins. Indeed, *Pedalium murex* contains several steroidal saponins notably diosgenin [10, 33]. *Pedalium murex* is used as substitute of *Tribulus terrestris* by many practitioners of Ayurvedic medicine to cure sexual diseases [34]. The features of renal damages induced by the ethanolic extract of *Tribulus terrestris* fruit (protodioscin) in rats were similar to our findings. These data led us to hypothesize that *P. murex* leaf and fruit aqueous extract nephrotoxicity could be linked to their steroidal saponins. Contrary to our findings, the ethanolic extract of *P. murex* fruit has showed protective effects against cisplatin-induced nephrotoxicity in Wistar rats [35, 36]. In addition, fruit aqueous and ethanolic extracts have exhibited protective effects against cadmium-induced nephrotoxicity in Wistar rats [36]. The divergent results could stem from the difference in the study length. Our study takes 28 days while prior study length at most 10 days. Furthermore, our data showed proteinuria in *P. murex-*treated rats without any change in creatinine and serum urea levels. Serum creatinine and urea are nonspecific and insensitive markers for chronic kidney diseases. They are the later indicator of renal impairment since their levels increase significantly only when more than 50% of the glomerular filtration rate is reduced [37]. Also, in glomerular nephrotic syndrome, proteinuria may occur without any increase in the creatinine level [38].

The activity of transaminases increased in response to hepatocellular damage. The markers of hepatocellular injury are ALT and AST, with ALT being the most specific [39]. In this study, the leafy stem extract decreased the AST activity at 200 mg/kg while the extract increased it at 400 mg/kg. The aqueous extract of *P. murex* leafy stem showed a significant elevation of the ALT level at 400 mg/kg and 800 mg/kg when compared to the control group. The increase in the ALT activity suggests hepatocyte necrosis or transient changes in their membrane permeability [40]. However, the protein level, an indicator of liver function, was not altered by the leafy stem extract even at the highest dose (800 mg/kg). Furthermore, liver histopathology did not show any sign of abnormalities. The aqueous extract of the fruit showed a nonconsistent pattern of variation of AST and ALT. Treatment with the highest dose of the fruit extract did not alter the AST, ALT, total protein levels, and liver histoarchitecture. These findings evidenced the lack of toxicological relevance of the variations in the ALT activity and the need to assess *P. murex* toxicity over an extended period.

The testis is a target organ of *P. murex* according to its uses in folk medicine [10]. The potential testicular toxicity of *P. murex* was evaluated via histopathology. Both extracts did not induce any deleterious effects on seminiferous tubule histoarchitecture. The effects of extracts on seminal parameters were not assessed in this study due to the fact that spermatogenesis lasts about 52 days in rats [41]. These preliminary data indicate that *P. murex* does not exhibit testicular toxicity after repeated dose 28-day of oral treatment in Wistar rats.

Considering our data, the aqueous extract of *P. murex* leafy stem and fruit seems to be safe at 200 mg/kg. Therefore, the no-observed-adverse-effect level (NOAEL) for *P. murex* leafy stem and fruit aqueous extracts is found to be 200 mg/kg.

## 5. Conclusion

The aqueous extracts from *P. murex* leafy stem and fruit do not present acute toxicity. However, both extracts should be used with caution when they are given over an extended period due to their potential nephrotoxicity. Studies over 90 days of exposure are needed to ensure the profile of safety and toxicity of *P. murex*.

## Figures and Tables

**Table 1:** Body weight gain and food intake of rats treated with the aqueous extracts of *P. murex* leafy stem (LS) and fruit (FR).

	Control	LS5000	FR5000
Initial body weight (g)	192.83 ± 7.97	201.17 ± 7.15	206.67 ± 6.85
Week 1 (g)	212.33 ± 4.33	220 ± 4.80	227 ± 4.27
Week 2 (g)	217 ± 3.46	223.5 ± 4.19	**233.33** **±** **2.32**^*∗*^
Body weight gain (%)	11.20 ± 2.28	10.04 ± 1.72	11.47 ± 2.09
Food intake (g/day)	54.18 ± 1.43	**66.82** **±** **1.55**^*∗*^	**64.86** **±** **1.58**^*∗*^

Values are expressed as the mean ± SEM. *p* ≤ 5% value was considered significant using Kruskal–Wallis test followed by Dunn's multiple comparison test. Asterisk denotes difference compared with the control group.

**Table 2:** Mean relative organ weight (%) of rats 14 days after single oral administration of the aqueous extracts of *P. murex* leafy stem (LS) and fruit (FR).

Organs	Control	LS5000	FR5000
Liver	4.21 ± 0.07	4.33 ± 0.06	4.23 ± 0.18
Kidneys	0.35 ± 0.02	0.37 ± 0.01	0.35 ± 0.01
Spleen	0.46 ± 0.03	0.38 ± 0.04	0.38 ± 0.04
Stomach	1.02 ± 0.08	1.06 ± 0.08	1.11 ± 0.06
Heart	0.36 ± 0.01	0.40 ± 0.04	0.34 ± 0.01
Lungs	0.89 ± 0.06	0.81 ± 0.03	0.75 ± 0.01

Values are expressed as the mean ± SEM (*n* = 3). *p* ≤ 5% value was considered significant using the Kruskal-Wallis test followed by Dunn's multiple comparison test.

**Table 3:** Mean body weight of rats after 28 days of treatment with the aqueous extracts of *P. murex* leafy stem (LS) and fruit (FR).

Days	Control	LS200	LS400	LS800	FR200	FR400	FR800
11	170 ± 1.78	167 ± 2.13	162 ± 2.35	172.6 ± 3.95	168 ± 2.97	172 ± 4.77	165 ± 3.75
7	192 ± 2.74	199 ± 1.85	188 ± 4.85	201.7 ± 4.32	188 ± 2.41	188 ± 6.35	185 ± 4.22
14	200 ± 2.82	208 ± 3.25	205 ± 5.25	217.6 ± 2.33	199 ± 2.62	195 ± 6.62	198 ± 6.70
21	211 ± 3.34	219 ± 2.77	216 ± 5.96	**234.1** **±** **4.86**^*∗*^	203 ± 3.07	195 ± 10.62	202 ± 6.60
28	222 ± 3.70	231 ± 4.41	220 ± 4.41	241.3 ± 5.12	210 ± 3.09	**198** **±** **12.92**^*∗*^	207.3 ± 7.66
Body weight gain (%)	23 ± 1.44	28 ± 1.87	26 ± 1.97	28 ± 1.36	20 ± 1.16	**12** **±** **4.46**^*∗*^	20 ± 3.55

Values are expressed as the mean ± SEM. ^*∗*^*p* ≤ 0.05 value was considered significant using ANOVA followed by Dunett's multiple comparison test. Asterisk denotes difference compared with the control group.

**Table 4:** Average food intake of rats after 28 days of treatment with the aqueous extracts of *P. murex* leafy stem (LS) and fruit (FR).

Days	Control	LS200	LS400	LS800	FR200	FR400	FR 800
7	119.6 ± 16.87	106.9 ± 2.79	94.93 ± 1.89	107.9 ± 4.88	100.9 ± 3.78	**88** **±** **2.10**^*∗*^	112 ± 3.46
14	107.7 ± 6.92	94.79 ± 1.99	94.36 ± 3.73	103.1 ± 1.86	100.3 ± 3.04	**85.36** **±** **3.72**^*∗*^	102.6 ± 6.86
21	90.29 ± 3.97	104.9 ± 7.15	95.57 ± 4.16	96.21 ± 4.95	92.57 ± 4.95	94.64 ± 5.87	95.5 ± 6.83
28	106.4 ± 4.28	108.4 ± 1.54	96.79 ± 2.78	104.8 ± 5.34	92.71 ± 4.40	**69.79** **±** **2.60**^*∗*^	91.93 ± 5.71

Values are expressed as the mean ± SEM. ^*∗*^*p* ≤ 0.05 value was considered significant using ANOVA followed by Dunett's multiple comparison test. Asterisk denotes difference compared with the control group.

**Table 5:** Relative organ weights (%) of rats after 28 days of treatment with the aqueous extracts of *P. murex* leafy stem (LS) and fruit (FR).

Organs	Control	LS200	LS400	LS800	FR200	FR400	FR800
Liver	3.83 ± 0.06	3.95 ± 0.18	3.96 ± 0.20	3.68 ± 0.06	3.76 ± 0.12	3.92 ± 0.28	3.51 ± 0.07
Kidneys	0.34 ± 0.01	0.39 ± 0.01	**0.42** **±** **0.02**^*∗*^	0.32 ± 0.01	0.35 ± 0.01	**0.41** **±** **0.03**^*∗*^	0.37 ± 0.01
Heart	0.35 ± 0.01	0.41 ± 0.01	0.38 ± 0.01	0.32 ± 0.01	0.37 ± 0.03	**0.43** **±** **0.03**^*∗*^	0.39 ± 0.02
Spleen	0.36 ± 0.00	0.42 ± 0.03	0.38 ± 0.01	0.33 ± 0.03	0.40 ± 0.01	0.37 ± 0.02	0.35 ± 0.02
Testis	0.56 ± 0.03	0.61 ± 0.04	0.60 ± 0.26	0.64 ± 0.02	0.59 ± 0.29	**0.67** **±** **0.01**^*∗*^	0.63 ± 0.01
Epididymis	0.33 ± 0.02	0.30 ± 0.01	0.33 ± 0.01	0.31 ± 0.01	0.33 ± 0.02	0.40 ± 0.02	0.34 ± 0.02

Values are expressed as the mean ± SEM. ^*∗*^*p* ≤ 0.05 value was considered significant using ANOVA followed by Dunett's multiple comparison test. Asterisk denotes difference compared with the control group.

**Table 6:** Urinalysis of rats after 28 days of treatment with aqueous extracts of *P. murex* leafy stem (LS) and fruit (FR).

Parameters	Groups						
	Control	LS200	LS400	LS800	FR200	FR400	FR800
*Occult blood (RBC/µL)*							
Neg	5	5	5	5	4	0	1
10	0	0	0	0	1	1	1
50	0	0	0	0	0	**4** ^*∗*^	**3** ^*∗*^

*Urobilinogen*							
Neg	5	5	5	5	5	5	5
+	0	0	0	0	0	0	0
++	0	0	0	0	0	0	0

*Ketone body*							
Neg	5	5	5	5	5	5	5
15	0	0	0	0	0	0	0

*Glucose (mg/dL)*							
Neg	5	5	5	5	5	5	5
50	0	0	0	0	0	0	0
1000	0	0	0	0	0	0	0

*Proteins (mg/dL)*							
Neg	5	5	0	0	3	1	0
30	0	0	1	0	1	0	0
100	0	0	1	1	1	**3** ^*∗*^	1
300	0	0	**3** ^*∗*^	**4** ^*∗*^	0	1	**4** ^*∗*^

*Nitrite*							
Neg	5	3	4	5	3	5	4
Pos	0	2	1	0	2	0	1

*Leukocytes*							
Neg	4	5	1	0	5	5	4
25	1	0	**3** ^*∗*^	1	0	0	1
70	0	0	1	**4** ^*∗*^	0	0	0

*pH*							
8	4	5	2	1	4	3	1
9	1	0	3	4	1	2	4

*Specific gravity*							
1.000	3	4	5	5	5	1	0
1.005	0	0	0	0	0	3	0
1.010	2	1	0	0	0	1	5

Volume (ml/24 h)^a^	5.38 ± 0.24	5.08 ± 0.25	5.22 ± 0.45	4.82 ± 0.45	5.02 ± 0.28	5.72 ± 0.22	5.70 ± 0.39

Values are expressed as the mean ± SEM. ^a^indicates the mean volume of the urine collected for each group. Asterisk denotes significant difference with control group.

**Table 7:** Biochemical parameters of rats after 28 days of treatment with aqueous extracts of *P. murex* leafy stem (LS) and fruit (FR).

Parameters	Control	LS200	LS400	LS800	FR200	FR400	FR800
AST (UI/L)	137.4 ± 4.24	**124** **±** **9.73**^*∗*^	**215.6** **±** **4.99**^*∗*^	134.9 ± 6.20	**117.6** **±** **3.17**^*∗*^	127.2 ± 3.45	131.4 ± 4.66
ALT (UI/L)	65.4 ± 1.63	65.2 ± 5.77	**94.9** **±** **9.46**^*∗*^	**96** **±** **9.04**^*∗*^	**80.2** **±** **5.03**^*∗*^	54 ± 3.61	59.6 ± 4.96
Glucose (mmol/L)	6.40 ± 0.18	**5.14** **±** **0.16**^*∗*^	**4.98** **±** **0.24**^*∗*^	**4.67** **±** **0.34**^*∗*^	5.97 ± 0.11	6.20 ± 0.28	5.57 ± 0.20
Triglycerides (mmol/L)	0.55 ± 0.06	0.46 ± 0.04	**0.86** **±** **0.06**^*∗*^	0.75 ± 0.12	0.53 ± 0.06	0.74 ± 0.08	**0.94** **±** **0.06**^*∗*^
Total cholesterol (mmol/L)	1.50 ± 0.11	1.91 ± 0.06	1.95 ± 0.07	1.97 ± 0.18	1.67 ± 0.07	1.73 ± 0.02	1.83 ± 0.12
Total protein (g/dL)	5.24 ± 0.19	6.11 ± 0.07	6.05 ± 0.11	6.63 ± 0.22	5.56 ± 0.14	6.29 ± 0.13	5.81 ± 0.08
Urea (g/L)	0.48 ± 0.04	0.49 ± 0.05	0.51 ± 0.05	0.56 ± 0.07	0.63 ± 0.02	0.61 ± 0.02	0.67 ± 0.04
Creatinine (mg/L)	6.6 ± 0.51	7 ± 0.63	6.2 ± 0.86	7.6 ± 0.51	6.6 ± 0.24	7.2 ± 0.58	7 ± 0.32
Sodium (mEq/L)	138.8 ± 1.28	139 ± 1.34	134 ± 1.91	138 ± 1.46	136.4 ± 1.36	137 ± 0.70	136.6 ± 0.98
Potassium (mEq/L)	4.1 ± 0.17	4.11 ± 0.17	4.19 ± 0.09	4.88 ± 0.24	3.82 ± 0.10	3.94 ± 0.12	4 ± 0.14
Chloride (mEq/L)	107 ± 2.10	109 ± 1.00	112.4 ± 1.25	112.2 ± 0.66	102.6 ± 0.68	106.2 ± 2.17	106.8 ± 1.39

The values are expressed as the mean ± SEM (*n* = 5). ^*∗*^*p* ≤ 0.05 value was considered significant using ANOVA followed by Dunnett's multiple comparison test. Asterisk denotes difference compared with the control group.

**Table 8:** Hematological parameters of rats after repeated treatment with aqueous extracts of *P. murex* leafy stem (LS) and fruit (FR).

Parameters	Control	LS 200	LS 400	LS 800	FR 200	FR 400	FR 800
RBC (T/L)	9.03 ± 0.33	8.54 ± 0.11	8.72 ± 0.12	8.04 ± 0.2	8.22 ± 0.24	8.73 ± 0.20	8.24 ± 0.14
HGB (g/dL)	14.78 ± 0.31	14.74 ± 0.1	15.76 ± 0.6	15.62 ± 0.25	13.4 ± 0.25	14.25 ± 0.44	13.63 ± 0.17
HCT (%)	47.41 ± 1.23	49.94 ± 0.95	47.6 ± 1.62	49.02 ± 0.96	42.92 ± 1.07	45.8 ± 1.49	43.15 ± 0.44
MCV (fL)	52.62 ± 0.85	58.52 ± 1.09	54.6 ± 1.28	61.14 ± 1.60	52.24 ± 0.40	52.27 ± 0.86	52.45 ± 1.08
MCH (g/dL)	16.24 ± 0.32	17.28 ± 0.15	18 ± 0.48	19.4 ± 0.35	16.32 ± 0.23	16.32 ± 0.28	16.58 ± 0.33
WBC (G/L)	18.98 ± 1.34	17.06 ± 1.33	16.72 ± 1.59	**12.74** **±** **0.78**^*∗*^	17.9 ± 2.23	13.73 ± 1.7	**11.98** **±** **0.58**^*∗*^
PLT (10^3^/*µ*L)	749 ± 91.28	**937.6** **±** **55.66**^*∗*^	**509.8** **±** **52.30**^*∗*^	**545.8** **±** **43.69**^*∗*^	770.4 ± 51.66	**976.25** **±** **32.80**^*∗*^	**974.5** **±** **44.71**^*∗*^

The values are expressed as the mean ± SEM (*n* = 5). ^*∗*^*p* ≤ 0.05 value was considered significant using ANOVA followed by Dunnett's multiple comparison test. Asterisk denotes difference compared with the control group.

## Data Availability

The majority of the data are included in this manuscript. Further data can be found from the corresponding author on reasonable request.

## References

[1] Agarwal A., Baskaran S., Parekh N. (2021). Male infertility. *The Lancet*.

[2] Blay R. M., Pinamang A. D., Sagoe A. E. (2020). Influence of lifestyle and environmental factors on semen quality in Ghanaian men. *International Journal of Reproductive Medicine*.

[3] Colpi G., Francavilla S., Haidl G. (2018). European Academy of Andrology guideline Management of oligo-astheno- teratozoospermia. *Andrology*.

[4] Duca Y., Calogero A. E., Cannarella R. (2018). Expert Opinion on Pharmacotherapy Current and emerging medical therapeutic agents for idiopathic male infertility Current and emerging medical therapeutic agents for idiopathic male infertility. *Expert Opinion on Pharmacotherapy*.

[5] Emokpae A., Uadia P. (2015). Male infertility in Nigeria: a neglected reproductive health issue requiring attention. *Journal of Basic and Clinical Reproductive Sciences*.

[6] Smits R. M., Mackenzie-Proctor R., Yazdani A., Stankiewicz M. T., Jordan V., Showell M. G. (2019). Antioxidants for male subfertility. *Cochrane Database of Systematic Reviews*.

[7] Showell M., Mackenzie-Proctor R., Yazdani A., Stankiewicz M., Hart R. (2014). Antioxidants for male subfertility (Review). *Cochrane Database of Systematic Reviews*.

[8] Tahvilzadeh M., Hajimahmoodi M., Toliyat T., Karimi M., Rahimi R. (2016). An evidence-based approach to medicinal plants for the treatment of sperm abnormalities in traditional Persian medicine. *Andrologia*.

[9] Marinho B. M., Guimaraes V. H. D., Sousa J. N. (2021). Brazilian Cerrado plant (arnica) Lychnophora ericoides Mart. (Asteraceae) toxicity characterization in mice. *Phytomedicine*.

[10] Devanesan A. A., Zipora T., G Smilin B. A., Deviram G., Thilagar S. (2018). Phytochemical and pharmacological status of indigenous medicinal plant Pedalium murex L.—a review. *Biomedicine and Pharmacotherapy*.

[11] Dossou-yovo K. M., Diallo A., Lawson-Evi P., Darré T., Bakoma B., Eklu-Gadégbéku K. (2020). Cytotoxicity, acute, and subacute study of hydroalcoholic root extract of Carissa spinarum L. On wistar rats. *Journal of Medicinal Food*.

[12] Patel D. K., Laloo D., Kumar R., Hemalatha S. (2011). Pedalium murex Linn.: an overview of its phytopharmacological aspects. *Asian Pacific Journal of Tropical Medicine*.

[13] Patel D. K., Kumar R., Prasad S. K., Hemalatha S. (2011). Pedalium murex Linn (Pedaliaceae) fruits: a comparative antioxidant activity of its different fractions. *Asian Pacific Journal of Tropical Biomedicine*.

[14] Dossou-agoin G. B., Gbankoto A., Azonbakin S., Osseni R., Yemoa A., Lalèyè A. (2021). Aqueous extract of Pedalium murex D. Royen ex L. leafy stem protects against lead induced testicular toxicity in Wistar rats. *Journal of Complementary and Integrative Medicine*.

[15] Sharma M., Arya D., Bhagour K., Gupta R. S. (2018). Modulatory effects of methanolic fruit fraction of Pedalium murex on sulphasalazine-induced male reproductive disruption. *Andrologia*.

[16] Balamurugan G., Muralidharan P., Polapala S. (2010). Aphrodisiac activity and curative effects of Pedalium murex (L.) against ethanol-induced infertility in male rats. *Turkish Journal of Biology*.

[17] Adebiyi O. E., Abatan M. O. (2013). Phytochemical and acute toxicity of ethanolic extract of Enantia chlorantha (oliv) stem bark in albino rats. *Interdisciplinary Toxicology*.

[18] Oecd (2002). Test N°. 423: acute oral toxicity-acute toxic class method. *Guidelines for the Testing of Chemicals*.

[19] Oecd (2018). Repeated dose 28-day oral toxicity study in rodents (OECD TG 407). *Guidance Document 150 on Standardised Test Guidelines for Evaluating Chemicals for Endocrine Disruption*.

[20] Sindete M., Gbankoto A., Osseni R. (2021). A 90-day oral toxicity study of an ethanolic root extract of Caesalpinia bonduc (L.) roxb. in wistar rats. *Evidence-based Complementary and Alternative Medicine*.

[21] Martins da Silva S. J. (2019). Male Infertility and antioxidants: one small step for man, no giant leap for andrology?. *Reproductive BioMedicine Online*.

[22] Mbow F. B., Diop I. (2019). Développer des approches pour comprendre, caractériser et adresser l’ infertilité et ses conséquences pour les individus et les familles en Afrique subsaharienne Le cas du Sénégal.

[23] Gad S. C. (2014). *Rodents Model for Toxicity Testing and Biomarkers*.

[24] Gomathi S., Sundaram R., Vijayabaskaran M., Kannan C., Sambathkumar R. (2017). Pedalium murex Linn leaves against LPS-induced oxidative stress, anxiety and depression behavioural alterations in rats. *Research Journal of Pharmacy and Technology*.

[25] Wu Y., Hu S., Yang D. (2022). Increased variation in body weight and food intake is related to increased dietary fat but not increased carbohydrate or protein in mice. *Frontiers in Nutrition*.

[26] Bello I., Bakkouri A. S., Tabana Y. M. (2016). Acute and sub-acute toxicity evaluation of the methanolic extract of Alstonia scholaris stem bark. *Medical Science*.

[27] Silva A. V., Norinder U., Liiv E., Platzack B., Öberg M., Törnqvist E. (2021). Associations between clinical signs and pathological findings in toxicity testing. *ALTEX*.

[28] Brigido H. P. C., Varela E. L. P., Gomes A. R. Q. (2021). Evaluation of acute and subacute toxicity of ethanolic extract and fraction of alkaloids from bark of Aspidosperma nitidum in mice. *Scientific Reports*.

[29] Hayes A. W., Kruger C. L. (2014). *Hayes’ Principles Methods and Methods of Toxicology*.

[30] Sellers R. S., Mortan D., Michael B. (2007). Society of Toxicologic Pathology position paper: organ weight recommendations for toxicology studies. *Toxicologic Pathology*.

[31] Lazic S. E., Semenova E., Williams D. P. (2020). Determining organ weight toxicity with Bayesian causal models: improving on the analysis of relative organ weights. *Scientific Reports*.

[32] Gosselin S., Ramaiah L., Earl L. (2009). Clinical chemistry in toxicity testing: scope and methods. *General, Applied and Systems Toxicology*.

[33] Abarikwu S. O., Onuah C. L., Singh S. K. (2020). Plants in the management of male infertility. *Andrologia*.

[34] Sharma V., Thakur M., Dixit V. K. (2012). A comparative study of ethanolic extracts of Pedalium murex Linn. fruits and sildenafil citrate on sexual behaviors and serum testosterone level in male rats during and after treatment. *Journal of Ethnopharmacology*.

[35] Tambade S., Ghadigaonkar S. (2021). Nephroprotective effect of ethanolic extract of Pedalium murex in cisplatin induced nephrotoxicity in wistar rats. *The Pharma Innovation Journal*.

[36] Shelke T., Kothai R., Adkar P. (2009). Nephroprotective activity of ethanolic extract of dried fruits of Pedalium murex linn. *Journal of Cell and Tissue Research*.

[37] Gandhi S., Srinivasan B. P., Akarte A. S. (2013). Potential nephrotoxic effects produced by steroidal saponins from hydro alcoholic extract of Tribulus terrestris in STZ-induced diabetic rats. *Toxicology Mechanisms and Methods*.

[38] Branten A. J. W., Vervoort G., Wetzels J. F. M. (2005). Serum creatinine is a poor marker of GFR in nephrotic syndrome. *Nephrology Dialysis Transplantation*.

[39] da Costa Araldi I. C., de Souza T. P., de Souza Vencato M. (2021). Preclinical safety assessment of the crude extract from Sida rhombifolia L. aerial parts in experimental models of acute and repeated-dose 28 days toxicity in rats. *Regulatory Toxicology and Pharmacology*.

[40] Diallo A., Eklu-gadegbeku K., Amegbor K. (2014). In vivo and in vitro toxicological evaluation of the hydroalcoholic leaf extract of Ageratum conyzoides L. (Asteraceae). *Journal of Ethnopharmacology*.

[41] Leblond C., Clermont Y. (1952). Definition of the stages of the cycle of the seminiferous epithelium in the rat. *Annals of the New York Academy of Sciences*.

